# Occurrence and subtyping of *Blastocystis* in coypus (*Myocastor coypus*) in China

**DOI:** 10.1186/s13071-021-05126-1

**Published:** 2022-01-06

**Authors:** Xuehan Liu, Fuzhen Ni, Rongjun Wang, Junqiang Li, Yaming Ge, Xuefeng Yang, Meng Qi, Longxian Zhang

**Affiliations:** 1 College of Animal Science and Veterinary Medicine, Henan Institute of Science and Technology, Xinxiang, 453003 Henan Province China; 2 Postdoctoral Research Base, College of Veterinary Medicine, Henan Agricultural University, Zhengzhou, 450046 Henan Province China; 3 College of Animal Science, Tarim University, Alar, 843300 Xinjiang Province China

**Keywords:** *Blastocystis*, Prevalence, Subtype, Zoonotic potential, Coypus

## Abstract

**Background:**

*Blastocystis* is an anaerobic unicellular protist frequently detected in the gastrointestinal tracts of humans and animals worldwide. However, the prevalence and subtype distribution of *Blastocystis* in the coypu (*Myocastor coypus*) population have not been reported so far. The aim of this study was to determine the prevalence, genetic characteristics, and zoonotic potential of *Blastocystis* isolates detected in coypus in China.

**Results:**

A total of 308 fecal samples were collected from coypus in seven regions across China and subsequently examined. *Blastocystis* was detected in 44 (14.3%) specimens by nested PCR amplification of the small subunit ribosomal rRNA (SSU rRNA) gene. Further DNA sequencing and phylogenetic analyses resulted in the identification of two zoonotic known subtypes, ST4 and ST5, and an unknown subtype. ST4 was the most predominant subtype observed in the samples. ST5 infections were only observed in three coypus. Factors that were associated with prevalence of *Blastocystis* included age, geographical region and subtype. Interestingly, this is the first report about a potentially novel subtype infecting coypus.

**Conclusions:**

This is the first comprehensive report of *Blastocystis* in *M. coypus* across a wide geographic range of China. A moderate degree of genetic divergence was observed. The presence of zoonotic subtypes in farmed *M. coypus* suggests that these animals have the potential to transmit blastocystosis to both humans and domestic animals. These findings provide a better understanding of the genetic diversity of *Blastocystis* in rodents and contribute towards the establishment of efficient blastocystosis control strategies in the investigated areas.

**Graphical abstract:**

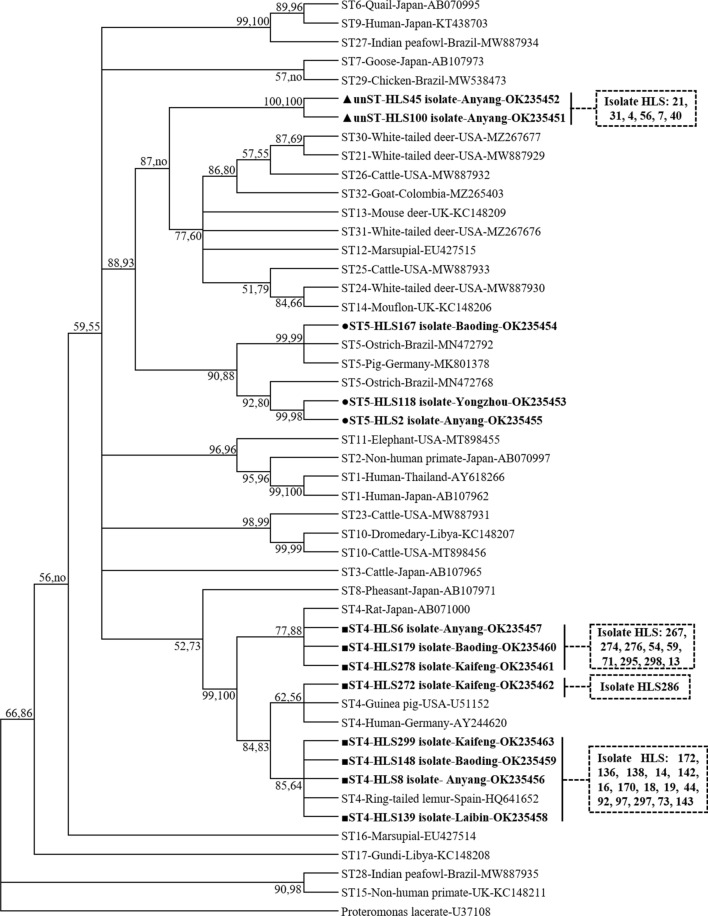

## Background

*Blastocystis* is a eukaryotic protozoan parasite that belongs to the stramenopile clade of heterokonts whose members are commonly observed in the intestinal tract of humans and animals worldwide [1, 2]. The pathogenicity of *Blastocystis* remains controversial because many infections produce subclinical and asymptomatic disease in humans and animals, but this parasite has been reported in patients with diarrhea and irritable bowel syndrome [2, 3]. The protist is believed to be transmitted via the fecal–oral route, involving direct contact with infected hosts and the ingestion of contaminated food or water [4, 5]. However, only a small number of published studies have reported zoonotic transmission between animals and their handlers [6]; therefore, a better understanding the zoonotic potential of *Blastocystis* is crucial. In addition, the identification of *Blastocystis* in a variety of pets, livestock and wildlife suggests widespread opportunities for these reservoir species to transmit the parasite to humans [7].

The genus *Blastocystis* exhibits a remarkable genetic diversity [8]. To date, a total of 32 *Blastocystis* subtypes have been reported based on polymorphisms within the small subunit ribosomal RNA (SSU rRNA) gene [4, 9, 10]. Subtypes ST1–ST32 have been described in various mammals and birds. However, based on the current criteria in place to qualify as a unique subtype, a total of 28 subtypes (ST1–ST17, ST21 and ST23–ST32) are generally widely recognized as being valid subtypes [4, 8–10]. In the past two decades, numerous epidemiologic studies on *Blastocystis* have demonstrated that most subtypes have a low host specificity. Infections have been associated with at least 14 subtypes (ST1–ST10, ST12, ST14, ST16 and ST24) in both humans and animals, indicating a strong potential for zoonotic transmission [11–13]. Moreover, > 95% of human *Blastocystis* cases have been attributed to ST1–ST4 [1, 2]. Thus, a variety of mammalian and avian hosts have been examined to determine the identity of *Blastocystis* subtypes carried by each species.

Wildlife, including animals in captivity, can serve as infection sources for zoonotic parasitic diseases [7]. Subtype analysis of *Blastocystis* infection in wildlife is important to improve our understanding of the potential role of wildlife in the transmission dynamics of these protozoa to livestock and human hosts [14, 15]. While a few studies have been published on *Blastocystis* in some rodents [6, 7, 13, 16], these studies did not specifically examine the prevalence of subtypes or conduct subtype distribution analysis of *Blastocystis* in the large semi-aquatic rodent *Myocastor coypus*. In the present study, we evaluated the prevalence and subtypes of *Blastocystis* in *M. coypus* across China, with the overall aim to better understand the risk of zoonotic spread of this parasite.

## Methods

### Source and sample collection

A total of 308 fecal samples were collected between August 2018 and March 2019 from seven *M. coypus* farms in Hebei Province (Baoding), Henan Province (Anyang and Kaifeng), Sichuan Province (Chengdu), Hunan Province (Yongzhou), Jiangxi Province (Ganzhou) and Guangxi Zhuang Autonomous Region (Laibin), in China (Table 1). Each farm possessed anywhere from 500 to 2000 *M. coypus*, and all had active breeding programs in place. Approximately 10–15% of animals representing each age group were randomly sampled at each farm. All fecal samples were collected immediately after excretion and placed in a 50-ml Eppendorf tube using disposable polyethylene gloves; the collection date and site, and the age and health condition of the animal were recorded at the time of fecal sample collection. All samples were immediately placed in a cooler with ice packs, transported to the laboratory within 48 h and stored at 4 °C until the time of analysis.

**Table 1:** Region and age distributions of *Blastocystis* subtypes in farmed *Myocastor coypus* in China

Variables	No. of farmed *M. coypus*	No. of subtypes			Total prevalence (% positive/total)	*P*-value	Odds ratio (95% confidence interval)
		ST4	ST5	Unknown ST			
Location							
Baoding, Hebei Province	35	3	1		11.4 (4/35)	0.347	2.839 (0.297–27.160)
Anyang, Henan Province	101	14	1	8	22.8 (23/101)	0.044	6.487 (0.829–50.759)
Kaifeng, Henan Province	52	10			19.2 (10/52)	0.093	5.238 (0.629–43.611)
Chengdu, Sichuan Province	40				0.0 (0/40)	0.184	–-
Yongzhou, Hunan Province	23		1		4.3 (1/23)	Reference	Reference
Ganzhou, Jiangxi Province	35				0.0 (0/35)	0.213	–-
Laibin, Guangxi Autonomous Region	22	6			27.3 (6/22)	0.034	8.250(0.903–75.414)
Age (months)							
< 3	65	8	2	6	24.6 (16/65)	Reference	Reference
3–6	47	8		1	19.1 (9/47)	0.494	0.725 (0.289–1.820)
> 6	196	17	1	1	9.7 (19/196)	0.003	0.329 (0.157–0.687)
Total	308	33	3	8	14.3 (44/308)		

*ST* Subtype

### DNA extraction and PCR analysis

Genomic DNA was extracted from 0.2 g of each fecal specimen using the E.Z.N.A® Stool DNA Kit, D4015-2 (Omega Bio-Tek Inc., Norcross, GA, USA), according to the manufacturer’s protocol, with minor modifications. Each DNA sample was stored at − 20 °C for subsequent molecular analysis.

Nested PCR amplification targeting the 479-bp fragment of the SSU rRNA gene was performed to screen for *Blastocystis* infection [17]. The primers RD3 (5′-GGGATCCTGATCCTTCCGCAGGTTCACCTAC-3′) and RD5 (5′-GGAAGCTTATCTGGTTGATCCTGCCAGTA-3′) that amplified a fragment of about 1780 bp were used in the primary PCR [18]. The primers Bla1 (5′-GGAGGTAGTGACAATAAATC-3′) and Bla2 (5′-TGCTTTCGCACTTGTTCATC-3′) that amplified a nested fragment of approximately 480 bp were used in the secondary PCR [17]. The PCR conditions were: 95 °C for 5 min; 35 cycles of denaturation at 95 °C/45 s, annealing at 54 °C or 65 °C/45 s and extension at 72 °C/1 min; with a final extension 72 °C for 8 min. Each PCR reaction was carried out in a total reaction volume of 25 μl containing 12.5 μl of 2× Taq PCR Master Mix (Sangon, Shanghai, China), 8.5 μl of deionized H_2_O, 1 μl of forward and reverse primers and 2 μl of genomic DNA or primary amplification products. Both positive (ST7 from quail) and negative (ultrapure H_2_O) controls were run in each PCR assay. All amplicons of the secondary PCR were resolved by electrophoresis in a 1% agarose gel with Golden View staining.

### Sequence and phylogenetic analyses

All positive PCR products were sequenced in both directions on an ABI 3730 DNA Analyzer (Applied Biosystems, Thermo Fisher Scientific, Foster City, CA USA). Sequencing reactions were performed by the Sangon Biotech Company (Shanghai, China). Raw sequences were corrected and assembled manually using the DNAStar 7.1 (https://www.dnastar.com/) and BioEdit 7.0 (http://www.mbio.ncsu.edu/BioEdit/bioedit.html) programs to guarantee the accuracy of the called nucleotides. To determine *Blastocystis* subtypes, each clean sequence was compared with GenBank sequences by BLAST (Basic Local Alignment Search Tool) analysis (http://www.ncbi.nlm.nih.gov/BLAST/) and the online platform of the definitions database of *Blastocystis* (https://pubmlst.org/bigsdb?db=pubmlst_blastocystis_seqdef), respectively. The reference sequences of known valid subtypes were downloaded from the GenBank database. All sequences obtained in this study were aligned with the reference sequences using Clustal X (http://www.clustal.org). A phylogenetic relationship was constructed by the neighbor-joining (NJ) method using the Kimura 2-parameter model and the maximum parsimony (MP) method with 1000 bootstrap replicates using the MEGA 7.0 software (http://www.megasoftware.net/).

Representative unique nucleotide sequences obtained herein have been submitted to the GenBank database under the accession numbers OK235451–OK235463.

### Statistical analysis

Differences in prevalence of *Blastocystis* subtypes between age groups and geographical locations were analyzed using a Chi-square (χ^2^) test and 95% confidence intervals (CIs) in the SPSS version 22.0 software package (www.ibm.com/products/spssstatistics). Differences were considered to be statistically significant at *P* < 0.05.

## Results and discussion

To the best of our knowledge, this study is the first report on the *Blastocystis* infection in *M. coypus* throughout China. Of the 308 fecal samples tested by nested PCR amplification of the SSU rRNA gene, 44 were positive for *Blastocystis* (prevalence: 14.3%; 95% Cl 10.4–18.2). Notably, the highest prevalence of *Blastocystis* was detected in fecal samples from *M. coypus* at the Laibin City farm (27.3%, 6/22), followed by those collected at the farms located in the cities of Anyang (22.8%, 23/101), Kaifeng (19.2%, 10/52), Baoding (11.4%, 4/35) and Yongzhou (13%, 3/23) (Table 1); in contrast, the parasite was not detected in any of the samples collected from farms in the cities of Chengdu and Ganzhou. In addition, higher infection rates were found at the Laibin and Anyang sites than at sites in the other five regions sampled, with the associations of infection rate with locality being statistically significant (*P* = 0.044 and 0.034) (Table 1). It has been reported that geographical and environmental factors might influence the prevalence of *Blastocystis* in animals and humans [5, 19]. For all locations sampled in the present study, significant differences in the observed prevalence may be attributable to different sanitation practices and standards between the farms.

When the data were stratified by age, prevalence of *Blastocystis* was found to be inversely related to age. The highest rate of infection was reported in animals aged < 3 months (24.6%, 16/65), followed those aged 3–6 months (19.1%, 9/47) and finally by those aged > 6 months (9.7%, 19/196) (Table 1). Evaluation of the correlation between age and infection rates based on the calculated odds ratios (ORs) and 95% CI values revealed a strong negative correlation, with an OR of 0.329 (95% CI 0.157–0.687; *P* = 0.003) associated with the > 6-month-old group. However, for the 3- to 6-month-old group,the correlation was statistically non-significant, with an OR of 0.725 (95% CI 0.289–1.820; *P* = 0.494) (Table 1). These findings are in contrast with those reported on *Blastocystis* infection in Chinese and Korean cattle [5, 20] as well as in young children in Turkey [21], all of which reported that age was significantly associated with *Blastocystis* infection.

Abundant studies on the molecular epidemiology of *Blastocystis* in livestock and wildlife have been published, many of which have chronicled transmission dynamics in addition to the zoonotic significance [7, 22]. However, the incidence of *Blastocystis* in rodents has not been sufficiently investigated, and only a few reports have been published to date [6, 7, 13, 16]. These published studies demonstrate the presence of *Blastocystis* infection in various rodent species in Belgium, Brazil, China, Colombia, France, Indonesia, Iran, Japan, Malaysia, Mexico, Poland, Romania, Singapore, Spain, Sweden, United Arab Emirate, UK and the USA, with the reported prevalence ranging from 3.13% to 100% [6, 7, 16, 23]. The overall *Blastocystis* prevalence of 14.3% (44/308) in *M. coypus* reported in the present study was consistent with prevalences reported in the Guinea pig (13.3%) from China and Polynesian rat (16.4%) from Indonesia [24, 25], but profoundly lower than those observed in brown rats in Malaysia (45.9%), in the water vole in the UK (36.5%), and in Sprague–Dawley rats in Turkey (61.1%) [26–28].

**Fig. 1:**
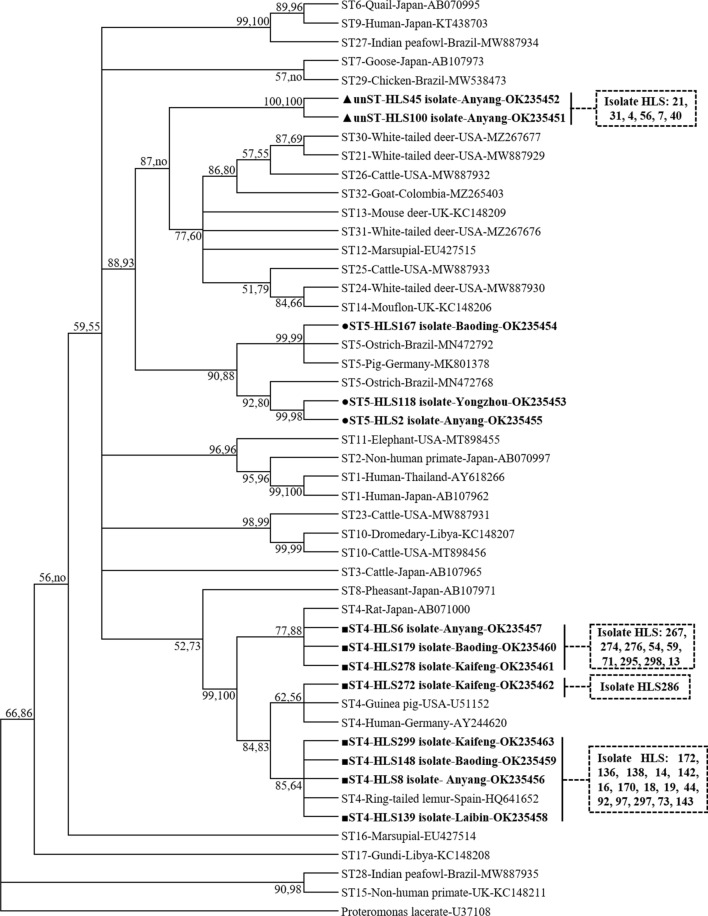
Phylogenetic relationship of the SSU rRNA genes (about 480 bp) of *Blastocystis* isolates from *Myocastor coypus* fecal samples. Relationships to other known *Blastocystis* subtypes were inferred by the MP and NJ methods based on evolutionary distances. Bootstrap values were obtained using 1000 pseudoreplicates, and values > 50% are shown. The filled triangles indicate potentially novel subtypes identified in the current study. *Proteromonas lacertae* was used as the outgroup for this tree. Abbreviations: no, not supported/lower bootstrap values

Genetic analysis of the 44 sequences obtained in this study confirmed the presence of two known subtypes of *Blastocystis*, ST4 and ST5. Remarkably, a previously unknown subtype (unST) was identified that shared a maximum nucleotide identity of only 92.4% with ST14, suggesting that the former is a potentially novel subtype (Fig. 1). Additional phylogenetic analysis corroborated the possible subtype identities, suggesting a moderate degree of genetic divergence compared to ST1–ST5 reported in other rodent species [7, 16]. The analysis of the subtype distribution in the 44 samples provides helpful information in the comparison of subtype prevalence in rodents from different geographic regions throughout the world. In our fecal samples, *Blastocystis* ST4 was the most predominant subtype, accounting for 75% (33/44) of subtypes in the analyzed samples; this proportion is similar to that reported in brown rats in Iran (60%) [23]. Also, the prevalence of ST4 was higher than that of any other ST in each age group of *M. coypus* examined (Table 1). For ST5 and unST in *M. coypus*, lower infection rates of 1% (3/308) and 2.6% (8/308) were observed, respectively. Each ST5 isolate was detected in three separate locations. Similarly, ST5 infections have been infrequently observed in the capybara and bank vole, accounting for a single case in each of the two species [22, 29]. However, the occurrence of the unST will require further study to validate its identification and its potential host specificity to *M. coypus*.

A number of published studies have clearly demonstrated that ST4 has a global distribution, with its detection in a broad array of host species, including rodents, suggesting a high degree of promiscuity [7, 20]. The number of studies on *Blastocystis* infection in rodents are far fewer than those in livestock. Interestingly, approximately 30 species of rodents have been surveyed to date, and at least 11 subtypes (ST1–ST8, ST10, ST13, ST15 and ST17) have been detected in rodent hosts [6, 7, 16, 23]. Of these, ST4 infection remains the most prevalent subtype, having been reported in > 17 rodent species, indicating that it has become well adapted to infect rodents (Table 2) [25, 27–38]. The zoonotic ST5 has only been detected sporadically in rodents (Table 2) [22, 28, 29], but has been detected in pigs in most provinces of China [39, 40]. Based on this observation, it would be interesting to examine whether or not transmission occurs between pigs and coypus. In addition, both ST4 and ST5 have been found to also infect cattle in China [20]. Hence, the infection risk to other animals around these farms cannot be ignored in view of the various transmission pathways of *Blastocystis.*

**Table 2:** Rodent hosts and geographic ranges of *Blastocystis* subtypes identified in this study

Subtype	Host (location)	References
ST4	Red squirrel (China, UAE, UK), Eastern chipmunk (China), Chinchilla (China), Guinea pig (China), Striped hamster (China), Sprague–Dawley rat (China, Turkey), Wistar rat (China), Spontaneous hypertensive rat (China), Patagonian mara (China), Brown rat (China, Japan, Malaysia, Iran, Spain), Flying squirrel (China), Norway rat (France), Rat (France, Japan, Singapore), Polynesian rat (Indonesia), Kangaroo rat (Mexico), Water vole (UK), Brazillian porcupine (USA), Coypu (China)	[25, 27–37]; This study
ST5	Bank vole (UK), Brown rat (Malaysia), Capybara (France), Coypu (China)	[22, 28, 29]; This study
unST	Coypu (China)	This study

*UAE* United Arab Emirate, *unST* Unknown subtype

Frequently detected in humans across a broad geographic range, ST4 is likely a significant concern for public health [11, 19, 41]. In contrast, infections with ST5 have been infrequently reported and only from a small number of countries [13, 42, 43]. Moreover, the potentially zoonotic ST4 and ST5 have been recently reported in patients in several provinces of China, including regions surveyed in the present study [42, 44–46]. Also notable it that transmission of *Blastocystis* between domesticated animals and their handlers has been demonstrated [6, 47]. Thus, there is a potential zoonotic threat to farmers/animal caretakers on *M. coypus* farms, but further study is required to determine the actual risk.

## Conclusions

This is the first molecular study of prevalence and subtype distribution of *Blastocystis* in *M. coypus*. The known zoonotic ST4 and ST5 subtypes as well as a potentially novel subtype were detected. These findings add to our current understanding of the genetic features of *Blastocystis* in rodent populations and of the potential zoonotic risk to farmers/animal caretakers of *M. coypus*. Further studies to extend the surveyed region and to analyze genetic characteristics of the unknown subtype are required.

## Data Availability

All data generated and analyzed during this study are included in the article as published. The representative unique nucleotide sequences obtained herein were submitted to the GenBank database under the accession numbers OK235451 through to OK235463.

## Abbreviations

BLAST: Basic local alignment search tool
MP: Maximum parsimony
NJ: Neighbor-joining
SSU rRNA: Small subunit ribosomal RNA
